# DUSP4 activates adipocyte thermogenesis via CRTC3 dephosphorylation-dependent UCP1 expression

**DOI:** 10.1038/s12276-026-01850-9

**Published:** 2026-10-02

**Authors:** Min Jeong Son, Hae Un Kook, Jaeeun Jung, Se-Jun Park, Young Joo Jeon, Kyoung-Jin Oh, Eun-Woo Lee, Baek Soo Han, Jang Hyun Choi, Kwang-Hee Bae, Won Kon Kim

**Affiliations:** 1 Metabolic Regulation Research Center, Korea Research Institute of Bioscience and Biotechnology (KRIBB), Daejeon, Republic of Korea; 2 Department of Functional Genomics, KRIBB School of Bioscience, Korea University of Science and Technology (UST), Daejeon, Republic of Korea; 3 Division of Biotechnology, College of Life Sciences and Biotechnology, Korea University, 145, Anam-ro, Seongbuk-gu, Seoul, Republic of Korea; 4 School of Pharmacy, Sungkyunkwan University, Suwon, Republic of Korea; 5 Biodefense Research Center, Korea Research Institute of Bioscience and Biotechnology (KRIBB), Daejeon, Republic of Korea; 6 Department of Biological Sciences, Ulsan National Institute of Science and Technology (UNIST), Ulsan, Republic of Korea

**Keywords:** Obesity, Fat metabolism

## Abstract

Obesity arises when a chronic energy surplus overwhelms the storage capacity of adipocytes and subsequently causes systemic metabolic dysfunction. Adaptive thermogenesis, primarily mediated by uncoupling protein 1 (Ucp1) in adipocytes, serves to counteract obesity by dissipating excess energy as heat. Despite extensive studies on thermogenic regulation, the phosphatases that regulate this protective metabolic pathway remain poorly understood. Here we identify dual-specificity phosphatase 4 (Dusp4) as a critical modulator of adipocyte thermogenesis via direct modulation of CREB-regulated transcription coactivator 3 (Crtc3). *Dusp4*-knockout mice exhibit impaired thermogenic capacity, diminished *Ucp1* expression, and increased susceptibility to diet-induced obesity coupled with severe insulin resistance. Mechanistically, Dusp4 directly dephosphorylates serine residues on Crtc3, facilitating its nuclear translocation and subsequent transcriptional activation of *Ucp1*. Depot-targeted restoration of catalytically active *Dusp4* in inguinal adipose tissue rescues local thermogenic gene expression and reinstates Crtc3 dephosphorylation-dependent *Ucp1* induction in *Dusp4*-knockout mice. Collectively, these findings identify Dusp4 as a key upstream phosphatase orchestrating the Crtc3–Ucp1 thermogenic axis, and highlight its potential as a therapeutic target for obesity-associated metabolic disorders.

**Figure:**
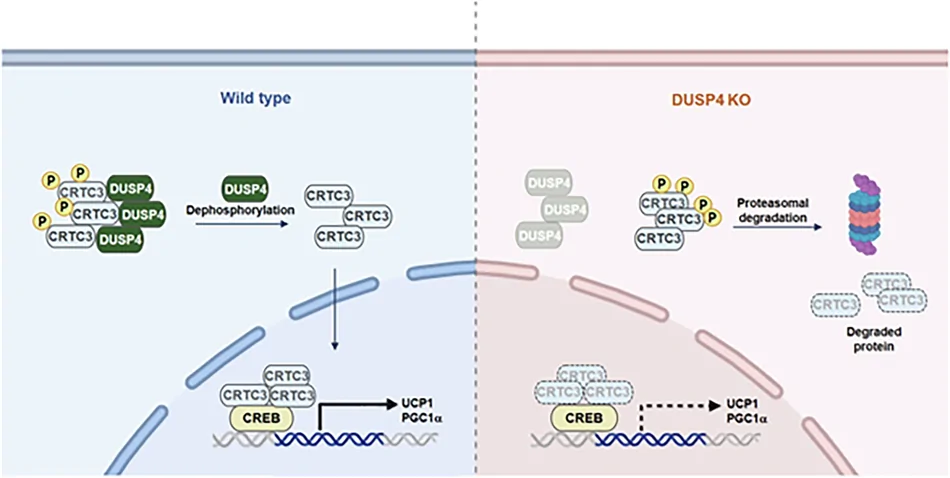

## Dusp4 phosphatase regulates adipocyte thermogenesis and obesity

Obesity, a global health crisis, is linked to metabolic disorders such as type 2 diabetes and cardiovascular disease. This study identifies dual-specificity phosphatase 4 (Dusp4) as a crucial regulator of thermogenesis in adipocytes, addressing its role in energy metabolism. The authors used genetically engineered mouse models to show that *Dusp4* deficiency impairs thermogenic gene expression, leading to increased susceptibility to obesity and insulin resistance. Dusp4 dephosphorylates the transcription coactivator Crtc3, promoting its nuclear translocation and activation of *Ucp1*, a key thermogenic gene. The restoration of *Dusp4* in adipose tissue rescues thermogenic function, highlighting its potential as a therapeutic target. These findings advance our understanding of adipocyte thermogenesis and suggest that enhancing Dusp4 activity could combat obesity by increasing energy expenditure.

This summary was initially drafted using artificial intelligence, then revised and fact-checked by the author.

## Introduction

Obesity has emerged as a global epidemic that is strongly associated with metabolic disorders such as type 2 diabetes, cardiovascular disease, immune dysfunction, and certain cancers. Chronic excess nutrient intake drives adipocyte hypertrophy and hyperplasia^1–3^, ultimately overwhelming the storage capacity of adipose tissue and disrupting systemic metabolic homeostasis^4,5^. By contrast, adaptive thermogenesis within adipose tissue acts as a compensatory mechanism to counteract obesity by converting surplus calories into heat^5–7^. Thermogenic activation, triggered by stimuli such as cold exposure or β-adrenergic agonists (e.g., CL316,243), critically depends on mitochondrial uncoupling protein 1 (Ucp1)^8–11^. Ucp1 uncouples oxidative phosphorylation (OXPHOS), dissipating the proton gradient across the mitochondrial inner membrane and generating heat instead of ATP synthesis, thereby increasing energy expenditure^12–15^. Thus, elucidating molecular mechanisms that regulate Ucp1 expression represents a promising therapeutic strategy for combating obesity and its associated metabolic dysfunctions.

Cellular signaling cascades orchestrate dynamic responses to environmental and metabolic stimuli^16–18^, governing critical cellular functions through tightly controlled phosphorylation–dephosphorylation cycles^19–22^. Among these signaling regulators, dual-specificity phosphatase 4 (Dusp4) has emerged as a versatile regulator implicated in various biological processes, including cell proliferation, immune responses, cancer progression, and metabolism^23–27^. Dusp4 primarily exerts its function by dephosphorylating key substrates, such as mitogen-activated protein kinases (MAPKs), ERK, p38, p300, and Smad4, thereby fine-tuning multiple signaling pathways and cellular outcomes^28–32^. However, despite extensive studies in other biological contexts, the involvement and role of Dusp4 in adipocyte metabolism^33,34^, particularly in thermogenic regulation, remain largely unexplored.

The CREB-regulated transcription coactivator 3 (Crtc3) is highly expressed in adipocytes and regulates energy metabolism through phosphorylation-dependent nuclear translocation^35,36^. However, the specific phosphatases regulating the phosphorylation state of Crtc3 have yet to be clearly identified. Given its adipose-enriched expression and enzymatic specificity, we hypothesized that Dusp4 regulates adipocyte thermogenesis by modulating Crtc3 activity, thereby promoting the transcriptional activation of *Ucp1*.

Here we identify Dusp4 as a critical regulator of adipocyte thermogenesis through direct dephosphorylation of Crtc3. Using genetically engineered mouse models, we demonstrate that loss of *Dusp4* impairs thermogenic gene expression, accelerates diet-induced obesity, and leads to systemic insulin resistance. Mechanistically, Dusp4 directly interacts with and dephosphorylates Crtc3, promoting its nuclear translocation and recruitment to the *Ucp1* promoter. Importantly, depot-targeted restoration of *Dusp4* in inguinal adipose tissue rescues the local thermogenic gene program in *Dusp4*-knockout (KO) mice, supporting an adipocyte-autonomous role for Dusp4 in adaptive thermogenesis. These findings establish the Dusp4–Crtc3–Ucp1 signaling axis as a novel regulatory pathway essential for adaptive thermogenesis and highlight Dusp4 as a viable therapeutic target to combat obesity and its metabolic complications.

## Materials and methods

### Cell culture and differentiation

Immortalized inguinal preadipocytes were kindly provided by Dr Shingo Kajimura (University of California, San Francisco). The inguinal preadipocytes were cultured in Dulbecco’s modified Eagle medium supplemented with 10% fetal bovine serum and 1% penicillin–streptomycin at 37 °C in a 5% CO_2_ incubator. For adipogenic differentiation, the cells were treated with isobutylmethylxanthine, dexamethasone, insulin, and rosiglitazone for 2 days. After differentiation induction, the medium containing insulin was replaced every 2 days. Mature inguinal adipocytes were treated with 1 µM norepinephrine (Sigma, A7257) for 5 h.

### Ectopic expression and knockdown of *Dusp4*

Mature inguinal adipocytes were induced to express *Dusp4* using a lentiviral system. Lentiviruses were produced in Lenti-X 293T cells (Takara, 632180) transfected with pLVX-green-*Dusp4* and the catalytically inactive *Dusp4*-CS mutant (Cys284Ser)^37^. After transfection, lentiviral particles were collected and filtered with 0.45-µm filters. The obtained *Dusp4* and *Dusp4*-CS mutant lentiviruses were concentrated using the Lenti-X Concentrator (Clontech, 631231). To achieve ectopic expression of wild-type (WT) *Dusp4* and *Dusp4*-CS mutant, mature inguinal adipocytes were infected with concentrated lentiviruses in the presence of polybrene (8 µg/ml). For *Dusp4* knockdown, we used a sequence based on the coding sequence from NCBI (NM_176933). The lentivirus vector or knockdown cassettes were co-transfected with the packaging vectors psPAX2 and pMD2.G in Lenti-X cells.

### Quantitative real-time PCR

Total RNA was extracted from cells using TRIzol reagent (Thermo Fisher Scientific, 15596018). cDNA was synthesized from 2 µg of total RNA using 10 pmol of random primers, dNTPs, reverse transcriptase, and RNasin^38,39^. The primers used for quantitative PCR (qPCR) were as follows: *Ucp1*, *Pgc1α*, *Pparγ*, *Prdm16*, *Cidea*, *Dio2*, *Atp5a*, *Cox2*, and *Cox4*. Gene expression levels were normalized to *L32* as the reference gene. qPCR reactions were performed using a CFX Maestro system (Bio-Rad) and 20× EvaGreen Supermix (Solgent, 31000-B500) according to the manufacturer’s instructions.

### Mitochondrial DNA copy number and respiratory chain gene expression

Total DNA was isolated from inguinal white adipose tissue (iWAT) or cultured adipocytes using an Exgene Cell DNA isolation kit (GeneAll). To determine the mitochondrial DNA (mtDNA) copy number, qPCR was performed using the mitochondrial gene *mt-Nd1* and normalized to the nuclear-encoded 18S rRNA. For gene expression analysis, qPCR primers were designed for representative OXPHOS subunits, including *Ndufa9, Sdha, Uqcrc2, Cox4, and Atp5a1*. All gene expression levels were normalized to L32.

### Western blot and immunoprecipitation

Total proteins were extracted using NP-40 lysis buffer (1% NP-40, 2.5 mM sodium phosphate, 1 mM EDTA, and 150 mM NaCl). A quantitative analysis of total protein concentration was performed using the Bradford protein assay (Bio-Rad, 5000002). For immunoblotting, equal amounts of protein lysates were incubated with primary antibodies against HSP90 (Santa Cruz, 1:5,000), DUSP4 (Santa Cruz, 1:1,000), UCP1 (Cell signaling, 1:1,000), PGC1α (Abcam, 1:1,000), FLAG (Sigma, 1:5,000), HA (Santa Cruz, 1:5,000), phospho-p38 (Cell signaling, 1:1,000), p38 (Cell signaling, 1:1,000), phospho-AMPK (Thr172) (Cell signaling, 1:1,000), total AMPK (Cell signaling, 1:1,000), phospho-CREB (Ser133) (Cell signaling, 1:1,000), and total CREB (Cell signaling, 1:1,000). For immunoprecipitation, cell lysates were incubated with an anti-CRTC3 antibody (Santa Cruz, 1:200) and protein A/G agarose beads (Calbiochem, IP05). The protein–antibody–bead complexes were washed three times with NP-40 lysis buffer. Protein samples were analyzed by western blotting. Phosphorylation signals were quantified by densitometry and normalized to total CRTC3 levels in the corresponding immunoprecipitation fractions (p-Ser (CRTC3)/ total CRTC3).

### Nuclear–cytoplasmic fractionation

Cytosolic and nuclear fractions were isolated using a commercial nuclear extraction kit (Thermo Fisher Scientific, NE-PER Nuclear and Cytoplasmic Extraction Reagents) according to the manufacturer’s instructions. Protein concentrations were determined by Bradford assay to ensure equal loading for subsequent analysis. Fraction purity was verified by immunoblotting for α-tubulin (as a cytosolic marker) and Parp (as a nuclear marker). Dusp4 and Crtc3 distributions were assessed by immunoblotting of each fraction.

### Luciferase assay

The luciferase assay was performed as previously described^38,39^. The pGL3-*Pgc1α* promoter plasmid was obtained from Addgene (Addgene, #8887). HEK293 cells were co-transfected with β-galactosidase expression plasmid to normalize transfection efficiency. The analysis used plasmids for the pGL4-*Ucp1* promoter region (−3.5 to +0.2 kb) and the pGL3-*Pgc1α* promoter region (−2.0 kb). Luciferase activity was measured 24 h after transfection using the Dual-Luciferase Reporter Assay System (Promega) according to the manufacturer’s protocol.

### Generation of stable *Dusp4*-overexpressing adipocytes using FACS

To generate stable *Dusp4*-overexpressing adipocytes, cells were transduced with GFP-tagged lentiviral *Dusp4* (or control virus) and GFP-positive cells were enriched by fluorescence-activated cell sorting (FACS). The sorted cells were expanded as preadipocytes and subsequently differentiated into mature adipocytes. *Dusp4* overexpression was confirmed by qPCR before downstream functional experiments.

### *Crtc3* overexpression and knockdown in the *Dusp4*-overexpressing background

Stable Dusp4-overexpressing adipocytes were transduced with lentiviruses expressing *Crtc3*, an shRNA targeting *Crtc3* (sh*Crtc3*), or matched controls. After transduction and differentiation, the mRNA levels of *Crtc3* and Ucp1 were quantified by qPCR, and mitochondrial respiration was assessed by Seahorse oxygen consumption rate (OCR) analysis as described in the following section.

### OCR measurement

Inguinal adipocytes were differentiated in Seahorse XF24-well plates and equilibrated in Seahorse XF assay medium containing 25 mM glucose, 0.5 mM pyruvate, and 2.5 mM L-glutamine for 1 hour at 37 °C before measurement. Thermogenesis was activated by treatment with 1 µM norepinephrine in the mature adipocytes. Seahorse assay was performed on inguinal adipocytes with oligomycin (1.5 μM), carbonyl cyanide m-chlorophenyl hydrazone (2 μM), antimyc/antimycin A (0.5/0.5 μM). The XF analyzers were used to measure the O_2_ consumption according to the suggested protocol (Agilent Technologies XF24)^39^.

### Isolation of mature adipocytes and stromal vascular fraction

Inguinal adipose tissue was minced and digested with collagenase in Krebs–Ringer buffer at 37 °C with gentle shaking. The digested suspension was filtered through a 250 μm nylon mesh and centrifuged at 500 × g for 5 min. Following centrifugation, the floating top layer containing mature adipocytes was collected, and the stromal vascular fraction (SVF) was recovered from the pellet. The floating mature adipocyte fraction was isolated and subjected to sequential packing through repeated centrifugation (500*g* for 5 minutes) until a pure, white adipocyte layer was obtained, effectively removing any remaining Krebs–Ringer buffer. Each fraction was obtained, and RNA was extracted for qPCR analysis. Fraction purity was validated by marker genes (*Pparγ* and *Ucp1* for mature adipocytes; *Cd3e* and *Cd68* for the immune-enriched SVF).

### *Dusp4-*KO mice

B6;129-Dusp4^*tm1Jmol*^/J mice (MGI:4456264) were obtained from the Jackson Laboratory (Bar Harbor). Exons 2 and 3 of the *Dusp4* gene were replaced with a neomycin resistance cassette in 129-derived embryonic stem cells. This strain was maintained on a mixed C57BL/6-129 genetic background by the donating laboratory. The mixed C57BL/6-129 genetic background mice were backcrossed for five generations onto the C57BL/6 strain. Mice were maintained in a specific pathogen-free animal facility. All animal procedures were approved by the Institutional Animal Care and Use Committee of the Korea Research Institute of Bioscience and Biotechnology and conducted in accordance with the Guide for the Care and Use of Laboratory Animals (US National Institutes of Health).

### Lentiviral delivery into iWAT and cold exposure

For depot-targeted reconstitution of *Dusp4* in vivo, concentrated lentivirus encoding *Dusp4* (Lenti-*Dusp4*) or control virus (Lenti-Ctrl) was injected directly into iWAT. Mice were anesthetized with isoflurane (Hana Pharm, Iflane) in oxygen; anesthesia was induced at 3–5% for 1–3 minutes. Using a 31 G insulin syringe, virus was slowly injected into two to three evenly distributed sites within the iWAT pad to minimize leakage and ensure broad depot coverage (total 90 μl per depot, ~30–40 μl per site). The needle was held in place for 1 minute after injection to prevent backflow. The viral titer used for all injections was matched across groups (1 × 10^8^ TU/ml titer as determined by functional transduction assay). The same injection volume and procedure were applied to all animals. Mice were then allowed to recover for 7 days before thermogenic challenge. For cold exposure, mice were housed at 6 °C for 7 days with ad libitum access to food and water, and tissues were harvested on day 7 immediately after cold exposure for histology, immunoblotting, and gene expression analyses. Where applicable, group allocation and downstream quantification were performed under standardized conditions to minimize experimental bias.

### Core body temperature measurement and infrared thermography

Core body temperature was assessed by measuring rectal temperature using a digital thermometer equipped with a lubricated rectal probe (Testo 925, Testo SE & Co. KGaA). Measurements were performed at the indicated time points during cold exposure under standardized conditions. Whole-body surface temperature was recorded using an infrared thermal imaging camera under controlled ambient conditions at a fixed distance. Thermal images were acquired on day 7 of cold exposure, and surface temperature was quantified from the defined region-of-interest using the manufacturer’s analysis software (FOTRIC 348A, FOTRIC).

### Indirect calorimetry and food intake measurements

Whole-body oxygen consumption (VO_2_), carbon dioxide production (VCO_2_), and energy expenditure were measured using an indirect calorimetry system (Oxylet Pro LE405, Panlab). Mice were acclimated to metabolic cages before data acquisition. Measurements were recorded continuously under specified ambient temperature conditions after cold exposure, with defined light/dark cycles. Energy expenditure was calculated from VO_2_ and VCO_2_ according to the manufacturer’s algorithm and normalized to body weight. Food intake was simultaneously monitored by the system.

### Statistical analysis

All quantitative data are presented as mean ± s.e.m. or mean ± s.d.. Statistical significance between two groups was determined by a two-tailed unpaired Student’s *t*-test, whereas comparisons among multiple groups were performed using one-way analysis of variance (ANOVA) followed by Tukey’s post hoc test. For longitudinal time-course experiments (e.g., body weight, rectal temperature, VO_2_ to VCO_2_ ratio), two-way repeated-measures ANOVA (group × time) with appropriate post hoc testing was used. In all cases, *P* < 0.05 was considered statistically significant.

## Results

### Thermogenic stimuli induce *Dusp4* expression in adipose tissue

**Fig. 1:**
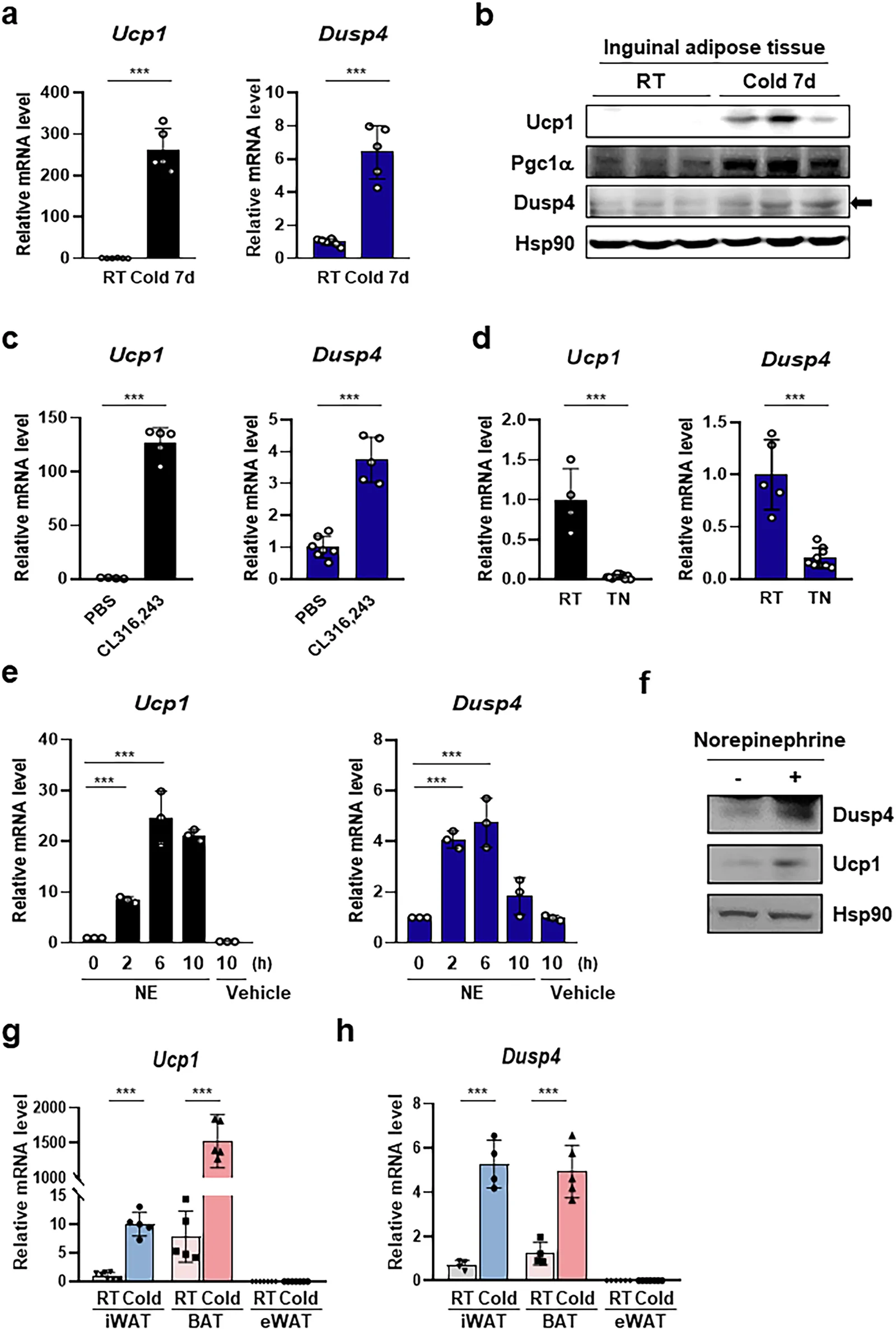
*Dusp4* expression is induced in adipose tissue by thermogenic stimuli. **a**, **b** Relative mRNA (**a**) and protein (**b**) expression levels of *Dusp4* in inguinal adipose tissue from mice exposed to cold (6 °C, 1 week). HSP90 was used as a loading control (*n* = 6–8 mice per group). **c** Expression of *Ucp1* and *Dusp4* mRNA in inguinal adipose tissue after daily injection of CL316,243 (1 mg/kg) intraperitoneally for 1 week (*n* = 5–7 mice per group). **d** Relative mRNA expression of *Ucp1* and *Dusp4* in inguinal adipose tissue from mice housed at room temperature (RT, 22 °C) or thermoneutrality (TN, 30 °C) for 1 week (*n* = 6–8 mice per group). **e**, **f** Relative mRNA (**e**) and protein (**f**) expression of *Ucp1* and *Dusp4* in mature inguinal adipocytes treated with norepinephrince (NE, 1 µM) for 5 hours (*n* = 3 independent experiments). **g**, **h** Relative mRNA expression of *Dusp4* (**g**) and *Ucp1* (**h**) in BAT, iWAT, and eWAT from mice housed at RT or exposed to cold (6 °C, 1 week). Data are presented as mean ± s.e.m. Statistical significance was determined by unpaired Student’s *t*-test (*P < 0.01, **P < 0.005, ***P < 0.0005).

To investigate whether Dusp4 is involved in adipose tissue thermogenesis, we first assessed its expression under thermogenic conditions. Indeed, thermogenic stimuli such as cold exposure and treatment with the β-adrenergic agonist CL316,243 robustly induced thermogenic markers, including Ucp1, in mouse inguinal adipose tissue (Fig. 1a–c). In parallel, both mRNA and protein levels of *Dusp4* were significantly upregulated in vivo (Fig. 1a, b). Conversely, under thermoneutral conditions (30 °C), which suppress thermogenic gene expression, *Dusp4* expression was markedly reduced (Fig. 1d). Consistent with the in vivo data, cultured inguinal adipocytes treated with norepinephrine to mimic cold stimulation showed markedly increased *Dusp4* mRNA and protein expression in vitro (Fig. 1e, f). Importantly, to define depot specificity, we quantified *Dusp4* and *Ucp1* across iWAT, brown adipose tissue (BAT), and epididymal adipose tissue (eWAT) after cold exposure. *Dusp4* was strongly induced in both iWAT and BAT, but minimally induced in eWAT; similarly, *Ucp1* was induced in iWAT and BAT, but remained negligible in eWAT (Fig. 1g, h). Notably, despite robust cold induction of *Dusp4* in BAT, *Dusp4* deficiency did not significantly blunt cold-induced *Ucp1* expression in BAT (Supplementary Fig. 5), indicating a depot-selective requirement for *Dusp4* that is most evident in inducible beige adipose tissue. To determine whether cold-induced *Dusp4* upregulation reflects an adipocyte-intrinsic response rather than changes in stromal/immune cell populations, we fractionated iWAT into mature adipocytes and SVF. The mature adipocyte fraction showed a robust induction of *Dusp4* on cold exposure, whereas SVF showed no appreciable induction; fraction purity was confirmed by enrichment of *Pparγ* and *Ucp1* in adipocyte fraction and *Cd3e* and *Cd68* in SVF (Supplementary Fig. 1). Together, these findings indicate that Dusp4 is a thermogenic stimulus-responsive factor that is dynamically induced in multiple adipose depots during thermogenic activation.

### Loss of *Dusp4* increases susceptibility to diet-induced obesity

**Fig. 2:**
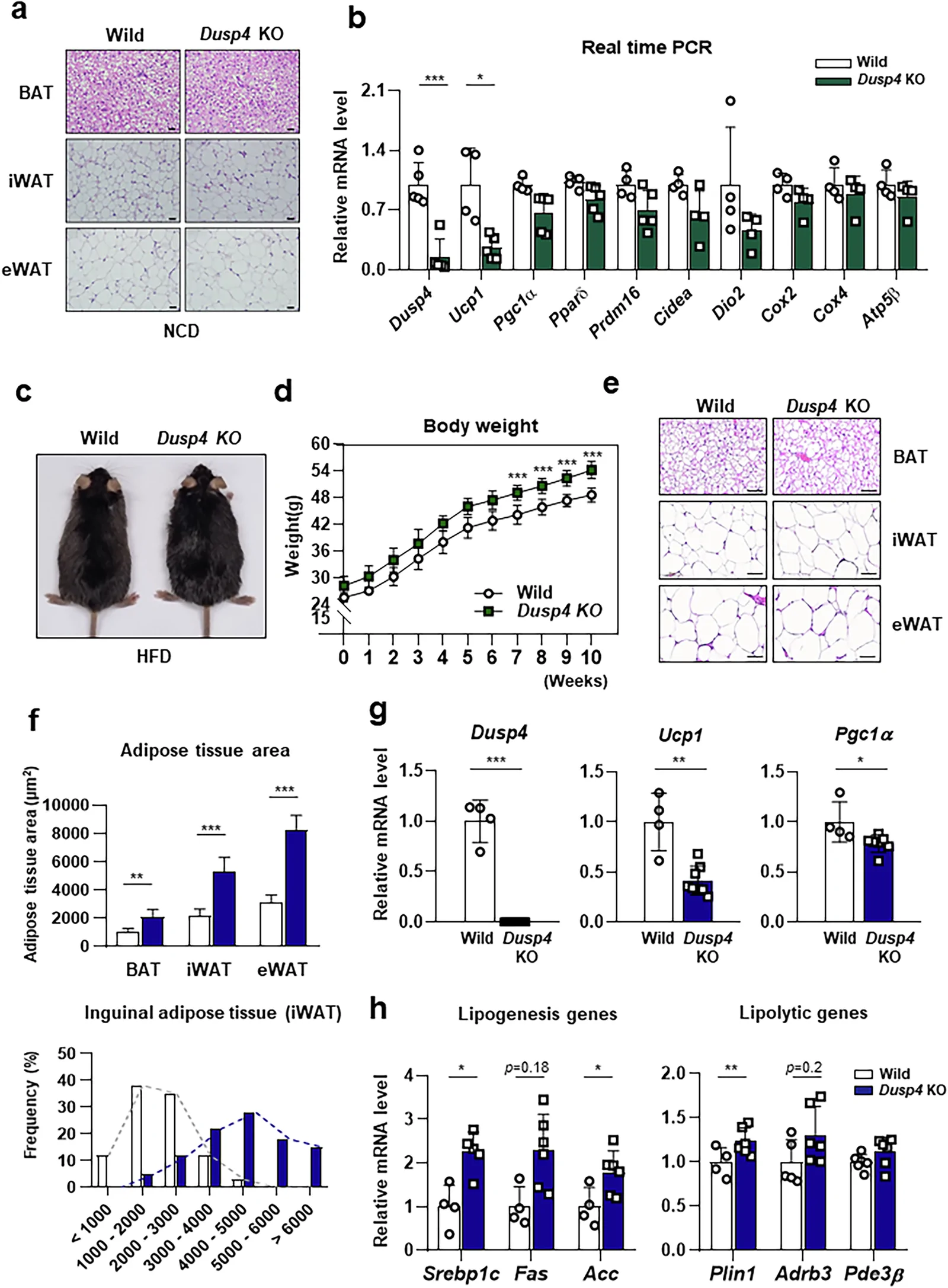
*Dusp4* deficiency impairs thermogenic gene induction and exacerbates diet-induced obesity. **a** Representative hematoxylin and eosin (H&E) staining of BAT, iWAT, and eWAT from WT and *Dusp4-*KO mice fed NCD (*n* = 6–7 mice per group). Scale bar, 60 μm. **b** Relative mRNA levels of thermogenic genes in inguinal adipose tissue from mice fed NCD (*n* = 7–8 mice per group). **c**, Representative whole-body images of WT and *Dusp4-*KO mice after 10 weeks of HFD. **d** Body-weight curves of WT and *Dusp4-*KO mice fed HFD for 10 weeks (*n* = 6–7 mice per group). **e** Representative H&E staining of BAT, iWAT, and eWAT from WT and *Dusp4-*KO mice fed HFD. Scale bar, 60 µm. **f** Quantification of adipocyte cross-sectional area in BAT, iWAT, and eWAT (upper), and adipocyte size distribution in iWAT (lower) from HFD-fed WT and *Dusp4-*KO mice. **g**, **h** Relative mRNA levels of thermogenic genes (**g**) and lipogenic genes (**h**) in inguinal adipose tissue after 10 weeks of HFD (n = 10 mice per group). Data are presented as mean ± s.e.m. Statistical significance was determined by unpaired Student’s *t*-test (*P < 0.01, **P < 0.005, ***P < 0.0005).

To determine whether *Dusp4* confers protection against obesity, we examined the metabolic phenotypes of *Dusp4-*KO mice under normal chow diet (NCD) and high-fat diet (HFD) conditions. On an NCD, *Dusp4*-KO mice exhibited no significant changes in adipose tissue mass and adipocyte size, but body weight was modestly higher than in WT controls (Fig. 2a and Supplementary Fig. 2a,b), indicating that baseline growth was not detectably altered under normal dietary conditions. Although the expression of thermogenic genes was modestly decreased (Fig. 2b), key genes involved in lipid metabolism, such as lipogenesis, β-oxidation, and lipolysis, were comparable between genotypes (Supplementary Fig. 2c–e). Glucose tolerance was modestly impaired, whereas insulin sensitivity remained normal (Supplementary Fig. 2f, g). Upon HFD challenge, however, *Dusp4-*KO mice showed markedly increased adiposity and body weight, as well as enlarged adipocytes, compared with WT mice (Fig. 2c–e). Quantitative morphometric analysis further confirmed adipocyte hypertrophy, showing increased adipocyte cross-sectional area across depots and a rightward shift in adipocyte size distribution within the iWAT of *Dusp4-*KO mice (Fig. 2f). The expression levels of key thermogenic genes, including *Ucp1* and *Pgc1α*, were substantially suppressed, whereas those of lipogenic genes such as *Srebp1c*, *Fas*, and *Acc* were elevated in *Dusp4-*KO mice (Fig. 2g, h). Although β-oxidation genes remained unchanged (Supplementary Fig. 3a), glucose tolerance and insulin sensitivity deteriorated substantially compared with WT controls (Supplementary Fig. 3b,c). Together, these results indicate that loss of *Dusp4* predisposes mice to diet-induced obesity by impairing thermogenic responses.

### *Dusp4* deficiency impairs thermogenic gene induction upon cold and β-adrenergic stimulation

**Fig. 3:**
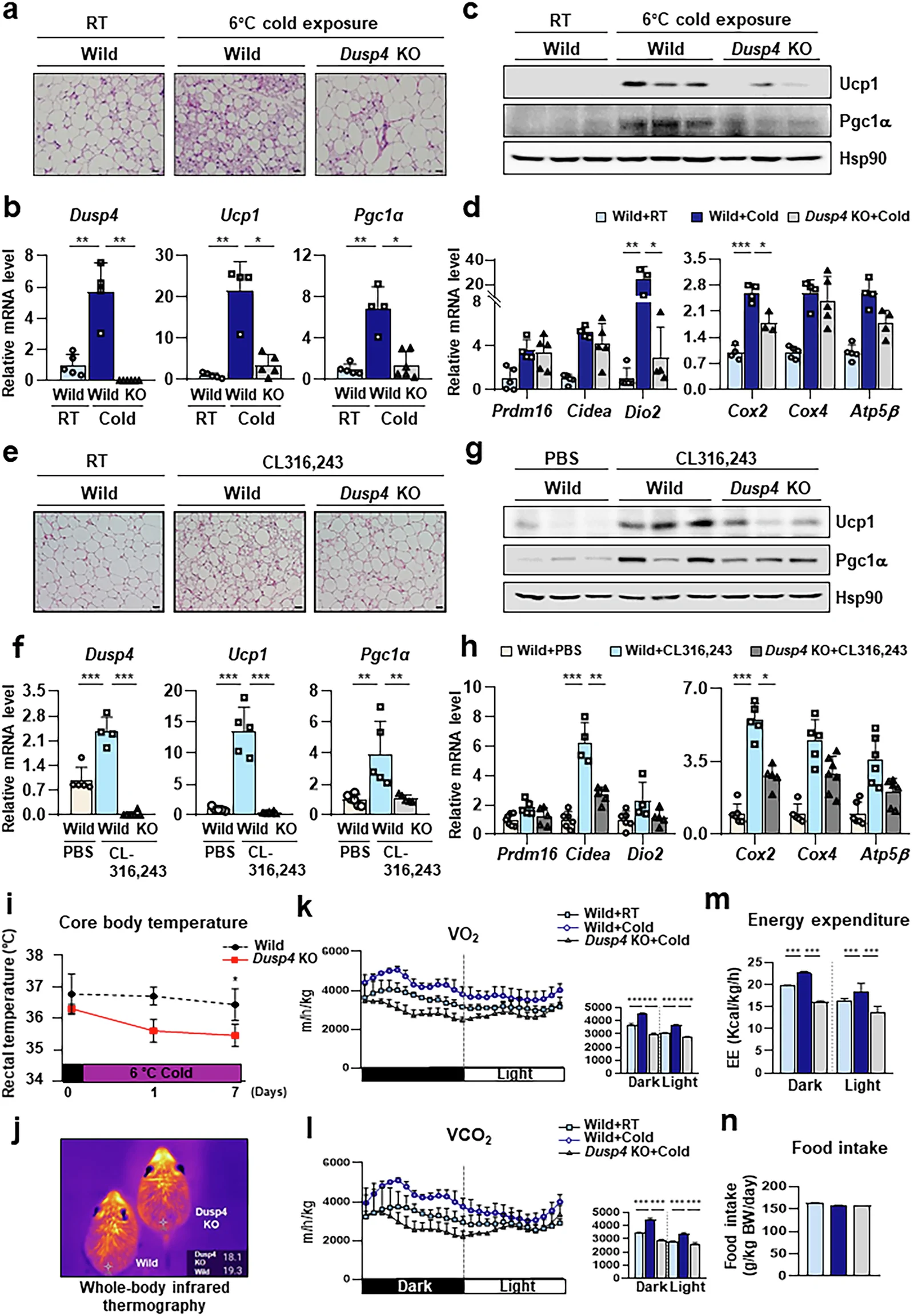
*Dusp4* is required for thermogenic activation in response to cold and β-adrenergic stimulation. **a** Representative H&E staining of inguinal adipose tissue from WT and *Dusp4-*KO mice exposed to cold (6 °C, 1 week). Scale bar, 60 µm. **b** Relative mRNA expression of *Dusp4*, *Ucp1*, and *Pgc1α* in inguinal adipose tissue after cold exposure (*n *= 8–10 mice per group). **c** Western blot analysis of Ucp1 and Pgc1α proteins in inguinal adipose tissue after cold exposure. Hsp90 was used as a loading control. **d** Relative mRNA levels of additional thermogenic genes in inguinal adipose tissue after cold exposure. **e** Representative H&E staining of inguinal adipose tissue from WT and *Dusp4-*KO mice after CL316,243 administration (1 mg/kg, 1 week). Scale bar, 60 µm. **f**, **g** Real-time qPCR analysis of *Dusp4*, *Ucp1*, and *Pgc1α* mRNA expression (**f**) and western blot analysis of protein expression (**g**) in tissue after CL316,243 administration. **h** Relative mRNA expression of thermogenic genes after CL316,243 administration (*n* = 7 mice per group). **i** Rectal temperatures of WT and *Dusp4-*KO mice measured at the indicated time points during cold exposure. **j** Whole-body infrared thermography of WT and *Dusp4-*KO mice on day 7 of cold exposure. **k** Whole-body VO_2_ measured by indirect calorimetry in WT + RT, WT + cold, and *Dusp4*-KO + cold mice; inset shows average VO_2_ during dark and light phases. **l** VCO_2_ measured by indirect calorimetry; inset shows averaged VCO_2_ during dark and light phases. **m** Energy expenditure (EE) calculated from VO_2_ and VCO_2_ during dark and light phases. **n** Daily food intake measured during the same period. Data are presented as mean ± s.e.m. Statistical significance was determined by one-way ANOVA with Tukey’s post hoc test (*P < 0.05, **P < 0.01, ***P < 0.001).

Reduced thermogenic capacity contributes significantly to obesity susceptibility. To elucidate the role of *Dusp4* in adaptive thermogenesis, we exposed *Dusp4-*KO mice to cold or CL316,243 stimulation. Upon cold challenge, *Dusp4-*KO mice exhibited larger lipid droplets and decreased expression of key thermogenic genes such as *Ucp1* and *Pgc1α* (Fig. 3a–d). Protein levels of these thermogenic factors were also substantially reduced (Fig. 3c). To assess mitochondrial remodeling in iWAT, we quantified mtDNA copy number and the expression of respiratory chain genes during cold challenge. The mtDNA copy number showed an increasing trend in the iWAT of WT mice, whereas this response was blunted in *Dusp4-*KO mice (Supplementary Fig. 6a). Consistently, multiple OXPHOS/respiratory chain transcripts *(Ndufa9, Sdha, Uqcrc2, Cox4, and Atp5a1)* and OXPHOS complex protein levels (CI–CV) were significantly reduced in *Dusp4-*KO iWAT, whereas mitochondrial biogenesis markers (*Tfam, Errα, Nrf1*) showed an attenuated cold-induced response in *Dusp4-*KO mice (Supplementary Fig. 6b–d). Similarly, after CL316,243 administration, adipocytes from *Dusp4-*KO mice showed greater lipid accumulation and markedly diminished induction of thermogenic genes and proteins compared with WT controls (Fig. 3e–h). Importantly, to assess functional thermogenesis, we measured core body temperature during cold exposure. *Dusp4-*KO mice showed a greater decline in rectal temperature and maintained a lower core temperature than WT controls throughout the cold challenge (Fig. 3i). Consistently, whole-body infrared thermography on day 7 revealed reduced surface temperature in *Dusp4-*KO mice (Fig. 3j). Consistent with these molecular defects, indirect calorimetry during cold exposure revealed that *Dusp4-*KO mice displayed reduced VO_2_ and VCO_2_ and a significant decrease in energy expenditure compared with WT mice under cold conditions (Fig. 3k–m), whereas food intake was comparable across groups (Fig. 3n). Collectively, these findings suggest that *Dusp4* deficiency impairs adaptive thermogenesis under physiological and pharmacological β-adrenergic stimuli, thereby exacerbating obesity susceptibility.

### Catalytic activity of Dusp4 is essential in promoting thermogenic gene expression in adipocytes

**Fig. 4:**
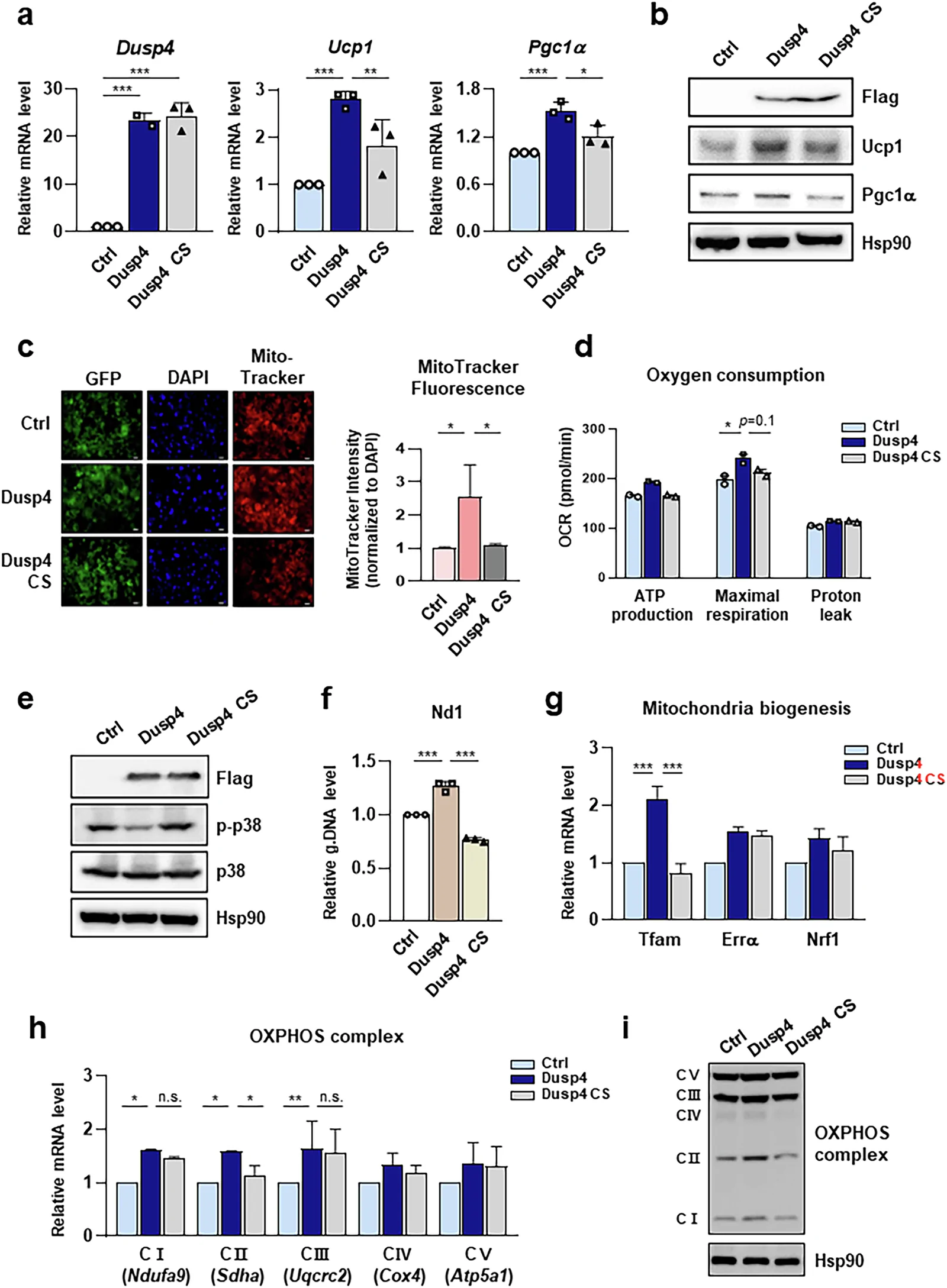
Catalytic activity of Dusp4 is essential for thermogenic gene activation via dephosphorylation of Crtc3. **a**, **b** Relative mRNA (**a**) and protein (**b**) expression levels of *Ucp1* in mature inguinal adipocytes transduced with lentiviruses expressing either WT *Dusp4* or the catalytically inactive *Dusp4*-CS mutant, followed by norepinephrine treatment (1 µM, 5 hours). **c** Representative fluorescence images (left) and quantification (right) of mitochondrial content (MitoTracker staining) in adipocytes transduced with *Dusp4* or *Dusp4*-CS mutant lentiviruses (*n* = 100 cells quantified from ≥3 independent experiments). Note the increased mitochondrial content in *Dusp4-*expressing cells. Scale bar, 50 µm. **d** OCR analysis in adipocytes transduced with indicated lentiviruses upon stimulation with norepinephrine (1 µM). **e** Immunoblot analysis of p-p38 and total p38 in cells expressing Flag-tagged *Dusp4* or *Dusp4*-CS, confirming loss of phosphatase activity in the CS mutant. Hsp90 was used as a loading control. **f** mtDNA copy number (*mt-Nd1* normalized to nuclear DNA) in mature adipocytes expressing Ctrl, *Dusp4*, or *Dusp4*-CS. **g** Relative mRNA expression of mitochondrial biogenesis markers (*Tfam*, *Erra*, and *Nrf1*). **h**, **i** Relative mRNA (**h**) and protein (**i**) levels of respiratory chain genes (*Ndufa9*, *Sdha*, *Uqcrc2*, *Cox4*, *Atp5a1*) in mature adipocytes expressing Ctrl, *Dusp4*, or *Dusp4*-CS after norepinephrine treatment (1 µM, 5 hours) (*n* = 3 independent differentiations/cell preparations). Data are presented as mean ± s.e.m. Statistical significance was determined by one-way ANOVA with Tukey’s post hoc test (*P < 0.05, **P < 0.01, ***P < 0.001).

To determine whether the phosphatase activity of Dusp4 is required for adipocyte thermogenesis, we ectopically expressed WT *Dusp4* or a catalytically inactive *Dusp4*-CS mutant in mature inguinal adipocytes using a lentiviral system. Treatment with norepinephrine robustly elevated *Ucp1* protein and mRNA levels in cells expressing WT *Dusp4*, whereas cells expressing the inactive *Dusp4*-CS mutant exhibited markedly impaired induction of thermogenic genes (Fig. 4a, b). Furthermore, mitochondrial mass, visualized by MitoTracker staining, was increased by *Dusp4* overexpression but remained suppressed in the *Dusp4*-CS mutant group (Fig. 4c). OCR, reflecting mitochondrial thermogenic activity, was markedly increased in response to norepinephrine in *Dusp4*-overexpressing cells but was compromised in cells harboring the *Dusp4*-CS mutant (Fig. 4d). Conversely, lentiviral knockdown of *Dusp4* mildly reduced thermogenic gene expression (Supplementary Fig. 4a,b). Importantly, to functionally validate the catalytic inactivity of the *Dusp4*-CS mutant, we assessed the phosphorylation of p38 MAPK. WT *Dusp4* reduced phospho-p38 (p-p38) levels without altering total p38, whereas *Dusp4*-CS failed to reduce p-p38 despite comparable expression levels (Fig. 4e). To quantitatively assess mitochondrial content, we measured the mtDNA copy number (*mt-Nd1* normalized to nuclear DNA) and found that *Dusp4* overexpression increased the mtDNA content, whereas *Dusp4*-CS failed to elicit this effect (Fig. 4f). Consistently, *Dusp4* induced a coordinated gene signature associated with mitochondrial biogenesis and the respiratory chain (*Ndufa9, Sdha, Uqcrc2, Cox4, and Atp5a1*), whereas the CS mutant did not fully recapitulate this program (Fig. 4g–i). These data firmly establish that the catalytic activity of *Dusp4* is required to enhance mitochondrial function and drive thermogenic gene expression in adipocytes.

### Dusp4 dephosphorylates Crtc3 to promote nuclear localization and transcriptional activation of thermogenic genes

**Fig. 5:**
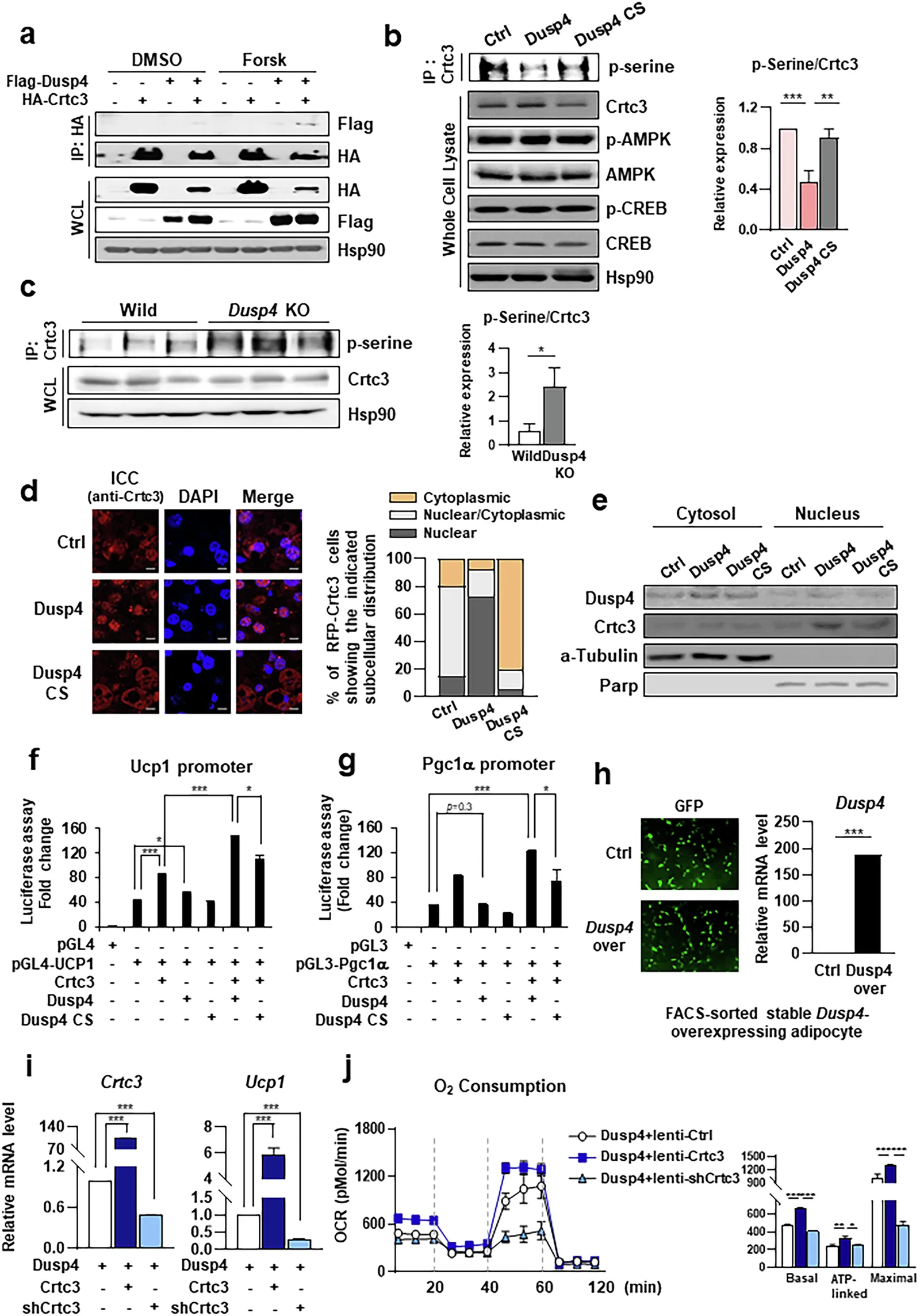
*Dusp4* dephosphorylates Crtc3 to promote nuclear localization and thermogenic gene transcription. **a** Co-immunoprecipitation (Co-IP) analysis of Flag-Dusp4 and HA-Crtc3 in HEK293 cells treated with forskolin (10 µM, 30 minutes), showing enhanced interaction upon stimulation. **b** IP and western blot analysis of Crtc3 phosphorylation status in mature inguinal adipocytes expressing *Dusp4* or catalytically inactive *Dusp4*-CS mutant (left). The p-serine (Crtc3)/Crtc3 ratio was quantified by densitometry (right). Whole-cell lysates from the same experimental conditions were additionally immunoblotted for phospho-AMPK (Thr172), total AMPK, phospho-CREB (Ser133), total CREB, and Hsp90 to evaluate upstream signaling pathways. **c** IP and western blot analysis of Crtc3 phosphorylation levels in inguinal adipocytes from *Dusp4*-KO mice (left). The p-serine (Crtc3)/Crtc3 ratio was quantified by densitometry (right). **d** Representative immunofluorescence images of Crtc3 localization in mature adipocytes expressing Ctrl, *Dusp4*, or *Dusp4*-CS (left). Quantification of Crtc3 subcellular distribution by scoring cells as nuclear, nuclear–cytoplasmic, or cytoplasmic across multiple independent fields (right; *n* ≥ 100 cells per condition). Scale bar, 5 µm. **e** Nuclear–cytosolic fractionation followed by immunoblotting for *Dusp4* and Crtc3. α-Tubulin and Parp were used as cytosolic and nuclear markers, respectively. **f** **g** Luciferase reporter assays driven by the *Ucp1* (**f**) and *Pgc1α* (**g**) promoters in HEK293 cells co-expressing Dusp4 and Crtc3 and treated with forskolin. **h** Verification of the stable *Dusp4*-overexpressing adipocyte model. Representative GFP fluorescence images (left) and relative mRNA expression of *Dusp4* (right) are shown for FACS-sorted cells. **i** Relative mRNA expression of *Crtc3* and *Ucp1* in *Dusp4*-overexpressing mature adipocytes transduced with lentiviral *Crtc3* (lenti-*Crtc3*) or sh*Crtc3*. **j** OCR measured by Seahorse analysis in *Dusp4*-overexpressing adipocytes after lentiviral-mediated overexpression or knockdown of *Crtc3* via sh*Crtc3* (*n* = 3 independent differentiations/cell preparations). Data are presented as mean ± s.e.m. Statistical significance was determined by one-way ANOVA with Tukey’s post hoc test (*P < 0.05, **P < 0.01, ***P < 0.001).

Having established a role for Dusp4 in adipocyte thermogenesis, we next explored its mechanistic basis. We hypothesized that Crtc3, a transcriptional coactivator regulated by phosphorylation-dependent nuclear translocation, may serve as a direct substrate of Dusp4. Co-immunoprecipitation assays in HEK293 cells co-expressing Flag-Dusp4 and HA-Crtc3 indicated that Dusp4 co-precipitates with Crtc3, and this interaction was enhanced by forskolin treatment, which mimics cold-induced cAMP signaling (Fig. 5a). *Dusp4* overexpression decreased phosphorylation levels of Crtc3, whereas the inactive *Dusp4*-CS mutant increased Crtc3 phosphorylation level (Fig. 5b). Importantly, this effect was not accompanied by global suppression of upstream kinase signaling: p-AMPK^Thr172^ and p-CREB^Ser133^ signals remained detectable in whole-cell lysates under the same conditions (Fig. 5b), supporting a phosphatase-layer mechanism for *Dusp4* in regulating Crtc3 phosphorylation state. Consistently, Crtc3 phosphorylation was elevated in *Dusp4-*KO adipose tissue (Fig. 5c). *Dusp4* overexpression promoted Crtc3 nuclear localization, as supported by immunofluorescence imaging and quantitative analysis across multiple cells (Fig. 5d), and independently confirmed by nuclear–cytosolic fractionation followed by immunoblotting (Fig. 5e). Nuclear-localized Crtc3 significantly enhanced luciferase activity driven by the *Ucp1* and *Pgc1α* promoters, an effect abrogated by the *Dusp4*-CS mutant (Fig. 5f, g). To establish Crtc3 as a functional mediator, we performed an epistasis experiment using FACS-sorted stable *Dusp4*-overexpressing adipocytes. *Crtc3* knockdown markedly blunted *Dusp4*-driven *Ucp1* induction, whereas *Crtc3* overexpression further enhanced *Ucp1* expression (Fig. 5h, i). Importantly, OCR measurements showed that *Crtc3* knockdown attenuated mitochondrial respiration in the *Dusp4*-overexpressing background, whereas Crtc3 overexpression increased OCR (Fig. 5j). Thus, *Dusp4* reduces inhibitory serine phosphorylation of Crtc3 to facilitate its nuclear translocation and transcriptional activation of key thermogenic genes.

### Adipose-specific restoration of *Dusp4* rescues impaired thermogenesis in vivo

**Fig. 6:**
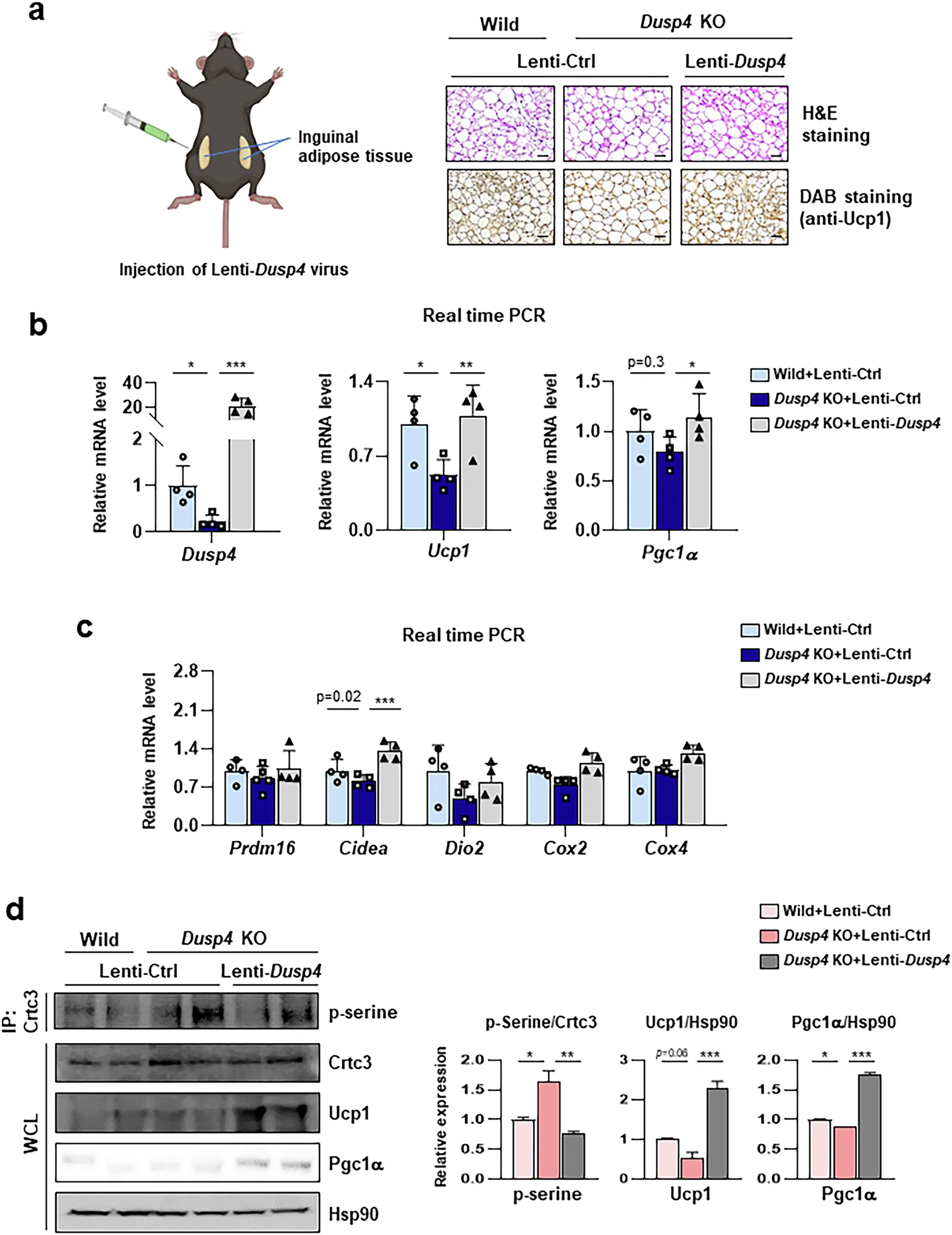
Adipose-specific restoration of *Dusp4* rescues thermogenic capacity. **a** Schematic diagram illustrating lentivirus-mediated restoration of *Dusp4* expression in iWAT (left). Representative H&E and immunohistochemistry (DAB staining; anti-Ucp1) images of inguinal adipose tissue after lentiviral injection (control vs. *Dusp4*) and cold exposure (right; *n* = 5 mice per group). Scale bar, 60 µm. **b**, **c** Relative mRNA expression of thermogenic genes, including *Ucp1* and *Pgc1α*, in iWAT after *Dusp4* restoration and subsequent cold exposure. **d** Immunoprecipitation followed by western blot analysis of phosphorylated serine residues (p-Ser), total Crtc3, Ucp1, and Pgc1α proteins in iWAT from virus-injected mice. Hsp90 was used as a loading control. Data are presented as mean ± s.e.m. Statistical significance was determined by one-way ANOVA with Tukey’s post hoc test (*P < 0.05, **P < 0.01, ***P < 0.001).

To test whether reintroducing *Dusp4* into *Dusp4-*KO mice could rescue impaired thermogenesis, we delivered lentiviral *Dusp4* into the inguinal adipose tissue of *Dusp4-*KO mice. *Dusp4* reconstitution markedly reduced lipid droplet accumulation and increased the expression of *Ucp1* and *Pgc1α* (Fig. 6a–d). Mechanistically, restoring *Dusp4* decreased the phosphorylation of Crtc3, enhancing its nuclear localization, and restored *Ucp1* expression to WT levels (Fig. 6d). Collectively, these findings demonstrate that depot-targeted reintroduction of *Dusp4* into iWAT is sufficient to restore the local thermogenic gene program in *Dusp4*-KO mice. Whether such depot-restricted rescue is sufficient to improve whole-body metabolic parameters will require adipose-wide delivery strategies and dedicated systemic phenotyping.

## Discussion

Obesity remains a global health crisis closely associated with metabolic dysfunction, yet therapeutic options targeting adipocyte thermogenesis to enhance energy expenditure are still limited. Currently available anti-obesity agents, such as Pparγ agonists (e.g., rosiglitazone) and natural compounds (e.g., resveratrol and docosahexaenoic acid), partially ameliorate metabolic disorders by inducing thermogenic responses in adipocytes^40–44^. Nevertheless, precise molecular targets enabling robust and selective activation of adipocyte thermogenesis are largely undefined, underscoring an urgent need to identify novel regulators that can effectively combat obesity through energy dissipation^45–47^.

In this study, we provide compelling evidence identifying *Dusp4* as a previously unrecognized but essential regulator of adipocyte thermogenesis and systemic metabolic homeostasis. Although several phosphatases have been implicated in metabolic regulation, the direct involvement of *Dusp4* in adipocyte thermogenic gene regulation represents a novel mechanism with important implications for the treatment of obesity. Our results reveal that *Dusp4* expression is selectively enriched in adipose tissue compared with other metabolic organs^23,45,48–51^, and notably induced under thermogenic conditions such as cold exposure and β-adrenergic stimulation. Cold exposure activates sympathetic outflow to adipose tissue, elevating norepinephrine/β-adrenergic signaling and downstream cAMP–PKA activity in adipocytes. This adrenergic axis engages CREB-dependent transcription and p38 MAPK signaling, which are established drivers of thermogenic gene induction and mitochondrial remodeling during cold adaptation^52^. In this context, *Dusp4* (also known as *Mkp2*)—encoded by a highly inducible dual-specificity phosphatase gene—may be transcriptionally upregulated as part of stimulus-responsive signaling programs and feedback control of MAPK pathways^53^. In addition, *Dusp4* has also been reported to be inducible in a cAMP-dependent manner in other cellular systems, supporting the plausibility that adrenergic cAMP signaling contributes to *Dusp4* induction during thermogenic activation^54^. Although we used a global KO model, our baseline analyses under NCD indicate that *Dusp4* loss does not overtly increase body weight; rather, the prominent metabolic divergence emerges under metabolic stress (HFD and thermogenic challenge), consistent with a role for *Dusp4* in supporting adaptive thermogenic and metabolic remodeling rather than general growth regulation. Conversely, depot-targeted restoration of *Dusp4* in iWAT rescued impaired thermogenic gene induction and reinstated a local thermogenic remodeling program, supporting *Dusp4* as a critical regulator in adipocytes. Because thermogenic adipose activation can influence systemic metabolic homeostasis^7,14,47^, future studies using adipose-wide rescue or scalable delivery will be needed to quantify impacts on whole-body glucose and lipid metabolism.

Mechanistically, our findings uncover a novel signaling axis in which *Dusp4* directly dephosphorylates Crtc3, promoting its nuclear translocation and consequently reinforcing a thermogenic/mitochondrial remodeling transcriptional module in adipocytes. Although Crtc3 is known to regulate adipocyte metabolism via its phosphorylation-dependent localization^55,56^, and can engage transcriptional programs beyond thermogenesis in a context- and stimulus-dependent manner^55,56^, the upstream phosphatases modulating its nuclear localization have remained elusive. Here we show that *Dusp4* interacts with Crtc3 and reduces its inhibitory serine phosphorylation (detected by pan–phospho-serine immunoblotting after Crtc3 immunoprecipitation), thereby shifting Crtc3 toward a nuclear-enriched state and enhancing transcriptional activation of the *Ucp1* and *Pgc1α* promoters, together with coordinated induction of thermogenic and respiratory chain gene signatures. Importantly, serine phosphorylation is a major regulator of CRTC family localization and activity, but it is not necessarily the sole determinant; Crtc3 nuclear competence can also be shaped by 14-3-3-dependent cytosolic retention, nucleocytoplasmic shuttling, protein stability/ubiquitin–proteasome control, and context-dependent protein–protein interactions that influence promoter engagement^36,55,56^. Accordingly, we interpret *Dusp4* as a phosphatase-layer gatekeeper that modulates one key axis of Crtc3 regulation during thermogenic stimulation, and acknowledge that these additional layers and potentially other Crtc3-controlled pathways may contribute depending on tissue context and stimulus.

The thermogenic remodeling in vivo is shaped not only by adipocyte-intrinsic transcriptional programs but also by tissue-level inputs, including sympathetic nerve activity, vascular remodeling, and noncoding RNA networks^57–60^. Consistent with prior studies implicating Crtc3 in broader thermogenic control^55,56^, these microenvironmental layers could modulate the amplitude and kinetics of Crtc3-dependent gene programs in a depot- and stimulus-specific manner^55–60^. In the present study, our data support a primary adipocyte-autonomous mechanism whereby *Dusp4* reduces inhibitory serine phosphorylation of Crtc3, promotes its nuclear localization, and enhances thermogenic transcription^58,59^. Nevertheless, *Dusp4* deficiency may secondarily alter innervation–vascularization states or microRNA programs (e.g., miR-206-linked regulatory modules), which may contribute to in vivo phenotypic variability^57,59,60^. Future studies integrating Crtc3 epistasis with readouts of sympathetic, vascular, and microRNA signatures will be important to distinguish *Crtc3*-dependent versus *Crtc3*-independent contributions to thermogenic regulation^60–63^.

The identification of the *Dusp4*ￚCrtc3 pathway significantly advances our understanding of the adipocyte thermogenic signaling network and opens promising avenues for therapeutic intervention. Given that *Dusp4* is enzymatically tractable, pharmacological strategies that enhance its catalytic activity or stabilize its interaction with Crtc3 could augment thermogenesis and improve metabolic outcomes. Such interventions may offer a novel strategy to enhance whole-body energy expenditure and reverse obesity-related metabolic dysfunction. Despite these significant findings, our study has several limitations warranting further exploration. First, our analysis was predominantly based on global *Dusp4-*KO mice; future studies using adipocyte-specific or inducible *Dusp4* deletion models will be necessary to delineate tissue-specific effects and minimize potential developmental compensatory mechanisms. Although key phenotypes were directionally consistent across independent experimental modalities, some assays were performed with relatively limited biological replicate numbers, which may reduce statistical power for select endpoints. Future studies with expanded cohorts, and where applicable contralateral paired injection designs within the same animal, will further refine effect size estimates, minimize inter-animal variability, and strengthen confidence in these conclusions. Second, although our data show that *Dusp4* reduces overall serine phosphorylation of Crtc3 and promotes its nuclear localization, the specific *Dusp4*-sensitive phosphorylation sites on Crtc3 remain unmapped. Future phospho-proteomic profiling coupled with site-directed mutagenesis will be required to pinpoint these residues and to distinguish direct from indirect effects. Finally, the translational relevance of our findings will require validation in human adipose tissue. We acknowledge the ongoing debate regarding the extent to which beige adipocytes and adaptive thermogenesis contribute to human metabolic regulation. Nonetheless, multiple studies have established that metabolically active BAT is present in adult humans and can be activated by cold exposure^61,62^; that BAT presence is associated with improved cardiometabolic health in large human cohorts^63^; and that β3-adrenergic stimulation (mirabegron) can increase BAT activity and improve metabolic parameters in humans^64^. These observations provide a rationale to interrogate the *DUSP4*–CRTC3 axis in human adipose datasets and cohorts. Future studies assessing *DUSP4* expression and its association with thermogenic signatures and metabolic traits in human adipose tissue will be essential to evaluate clinical feasibility.

In conclusion, we establish *Dusp4* as a key regulator of adipocyte thermogenesis through direct modulation of Crtc3 phosphorylation. This novel *Dusp4*–Crtc3–*Ucp1* signaling axis provides a mechanistic foundation for adaptive thermogenesis and highlights *Dusp4* as a promising therapeutic target for combating obesity and its associated metabolic disorders by enhancing energy expenditure.

## Supplementary information

Supplementary Information
