# Supplementary material for: DUSP4 activates adipocyte thermogenesis via CRTC3 dephosphorylation-dependent UCP1 expression
Source: Exp Mol Med. 2026 Oct 2;58(10):3382–94. doi: 10.1038/s12276-026-01850-9 (PMC13737205; doi:10.1038/s12276-026-01850-9)
Supplement: Supplementary file 1 [file 12276_2026_1850_MOESM1_ESM.pdf]

DUSP4 activates adipocyte thermogenesis via Crtc3 dephosphorylation-dependent UCP1 expression

Min Jeong Son[1], Hae Un Kook[1], Jaeeun Jung[1,2], Se-Jun Park[3], Young Joo Jeon[1], Kyoung-Jin Oh[1,2], Eun-Woo Lee[1,2], Baek Soo Han[2,4], Jang Hyun Choi[5,*], Kwang-Hee Bae[1,**] and Won Kon Kim[1,2,***]

[1]Metabolic Regulation Research Center, Korea Research Institute of Bioscience and Biotechnology (KRIBB), Daejeon 34141, Republic of Korea

[2]Department of Functional Genomics, KRIBB School of Bioscience, Korea University of Science and Technology (UST), Daejeon 34141, Republic of Korea

[3]Division of Biotechnology, College of Life Sciences and Biotechnology, Korea University, 145, Anam-ro, Seongbuk-gu, Seoul 02841, Republic of Korea

[4]Biodefense Research Center, Korea Research Institute of Bioscience and Biotechnology (KRIBB), Daejeon 34141, Republic of Korea

[5]Department of Biological Sciences, Ulsan National Institute of Science and Technology (UNIST), Ulsan 44919, Republic of Korea

Address correspondence to:

[*]Jang Hyun Choi, Department of Biological Sciences, Ulsan National Institute of Science and Technology (UNIST), Ulsan 44919, Republic of Korea. Phone: 82-52-217-2543; email: janghchoi@unist.ac.kr;

[**]Kwang-Hee Bae, Metabolic Regulation Research Center, Korea Research Institute of Bioscience and Biotechnology (KRIBB), Daejeon 34141, Republic of Korea. Phone: 82-42-860-4268; email: khbae@kribb.re.kr;

[***]Won Kon Kim, Metabolic Regulation Research Center, Korea Research Institute of Bioscience and Biotechnology (KRIBB), Daejeon 34141, Republic of Korea. Phone: 82-42-860-4265, email: wkkim@kribb.re.kr

Material and method

Animal experiments

B6;129-*Dusp4*^{tm1Jmol}/J mice (MGI:4456264) were obtained from the Jackson Laboratory (Bar Harbor, ME, USA). This strain was generated using 129-derived embryonic stem cells in which exons 2 and 3 of the Dusp4 gene were replaced with a neomycin resistance cassette. The donating laboratory maintained the mice on a mixed C57BL/6;129 genetic background, and the line was further backcrossed to the C57BL/6 strain for five generations to standardize the genetic background. All animals were housed in a specific pathogen-free facility. All procedures were reviewed and approved by the Institutional Animal Care and Use Committee of the Korea Research Institute of Bioscience and Biotechnology (KRIBB) and were conducted in accordance with the Guide for the Care and Use of Laboratory Animals of the U.S. National Institutes of Health.

Quantitative real-time PCR

Total RNA was isolated from cells using TRIzol reagent (Thermo Fisher Scientific, 15596018). cDNA was synthesized from 2 µg of RNA using random primers (10 pmol), dNTPs, reverse transcriptase, and RNasin. Quantitative PCR was performed with primers targeting lipogenic genes (*Srebp1c*, *Acc*, *Fas*), lipolytic genes (*Plin1, Adrb3*, *Pde3 β*), and β-oxidation genes (*F0f1atpase*, *Accβ, Cpt1β, Mcad*). Gene expression levels were normalized to *L32* as the reference gene. qPCR assays were conducted on a CFX Maestro system (Bio-Rad, CFX Maestro) using 20x EvaGreen Supermix (Solgent, 31000-B500) according to the manufacturer's instructions.

Western blot

Total proteins were extracted using NP-40 lysis buffer (1% NP-40, 2.5 mM sodium phosphate, 1 mM EDTA, and 150 mM NaCl). A quantitative analysis of total protein concentration was performed using the Bradford protein assay (Bio-Rad, 5000002). Cell lysates were incubated with primary antibodies

for Hsp90 (Santa Cruz, 1:5,000), Dusp4 (Santa Cruz, 1:1,000), and Flag (Sigma, 1:5,000). Following primary antibody incubation, membranes were washed and probed with secondary antibodies (Santa Cruz, 1:2,000). Signals were detected using an enhanced chemiluminescence (ECL) system (GE Healthcare, RPN2232).

Supplementary Figures

Supplementary Fig. 1

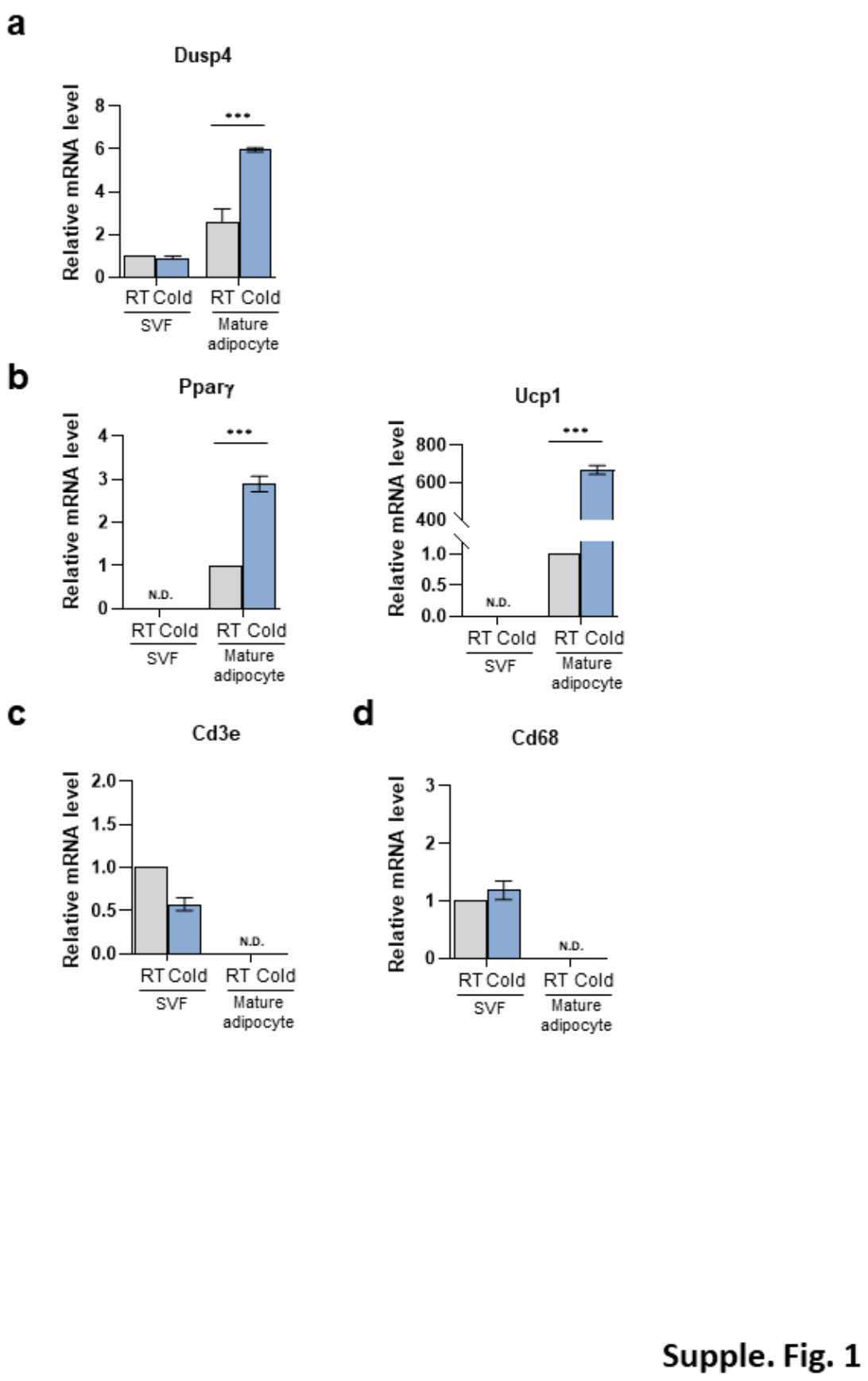

Supplementary Fig. 1 Cold-induced Dusp4 upregulation is enriched in mature adipocytes compared with the SVF. a Relative mRNA expression of *Dusp4* in SVF and mature adipocyte fractions isolated from the iWAT of mice housed at room temperature (RT) or exposed to cold. **b** Validation of fraction purity using mature adipocyte markers Pparγ and Ucp1 (N.D., not detected in SVF). **c, d** Immune cell markers Cd3e (T cell marker; **c**) and Cd68 (macrophage marker; **d**) are detected in SVF but not in mature adipocytes (N.D., not detected in mature adipocytes), confirming minimal immune contamination of the adipocyte fraction. Data are presented as mean ± SEM. Statistical significance was determined using unpaired two-tailed Student's *t*-test. ****p* < 0.0005.

Supplementary Fig. 2

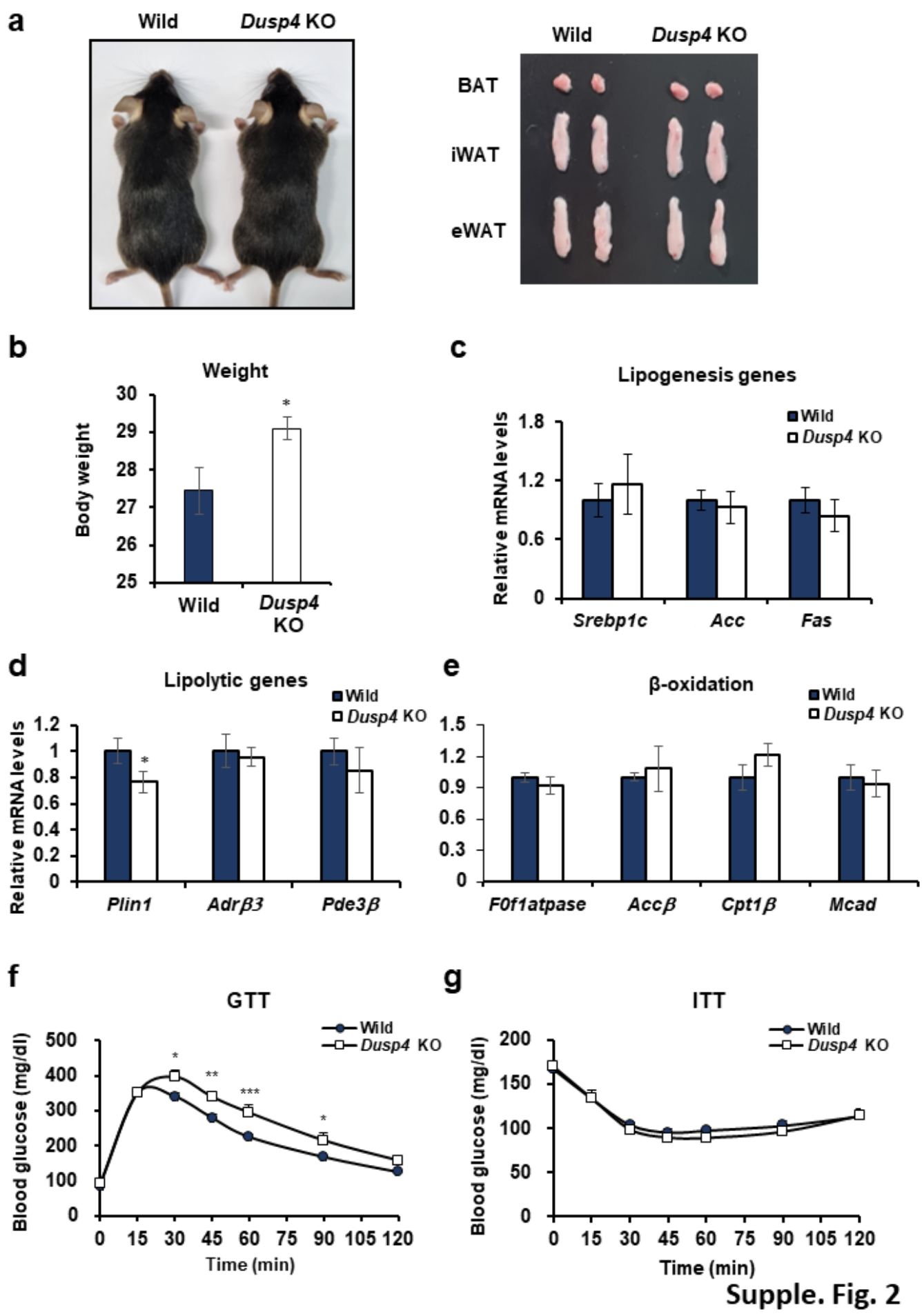

Supplementary Fig. 2 Metabolic phenotype of *Dusp4* knockout mice under normal chow diet (NCD). To assess the baseline metabolic characteristics of *Dusp4*-deficient mice in the absence of dietary challenge, *Dusp4* knockout (KO) and wild-type (WT) mice were maintained on a normal chow diet (NCD) and subjected to morphological, molecular, and metabolic evaluations. **a** Representative images of whole-body morphology (left) and dissected adipose tissues (right) from WT and *Dusp4* KO mice revealed no gross anatomical differences under NCD conditions. **b** Body weight measurements showed a modest but statistically significant increase in body weight in Dusp4 KO mice compared with WT controls (n = 5–6 per group). **c**–**e** Quantitative real-time PCR analyses of inguinal white adipose tissue (iWAT) demonstrated a modest reduction in lipogenic gene expression in KO mice (**c**), while the

expression of genes involved in lipolysis (**d**) and β-oxidation (**e**) remained largely unchanged compared to WT controls. **f**, **g** *Dusp4* KO mice exhibited significantly impaired glucose clearance during the glucose tolerance test (GTT) (**f**), while the insulin tolerance test (ITT) (**g**) revealed no notable changes. These findings indicate that under normal chow diet, *Dusp4* KO mice exhibit largely normal metabolic phenotype, with only subtle impairments in thermogenic gene expression and glucose homeostasis, suggesting that the protective role of DUSP4 becomes more prominent under metabolic stress such as high-fat diet. Data are presented as mean ± SEM. Statistical comparisons were made using two-tailed unpaired Student's *t*-test; *$p<0.01$, **$p<0.005$, ***$p<0.0005$.

Supplementary Fig. 3

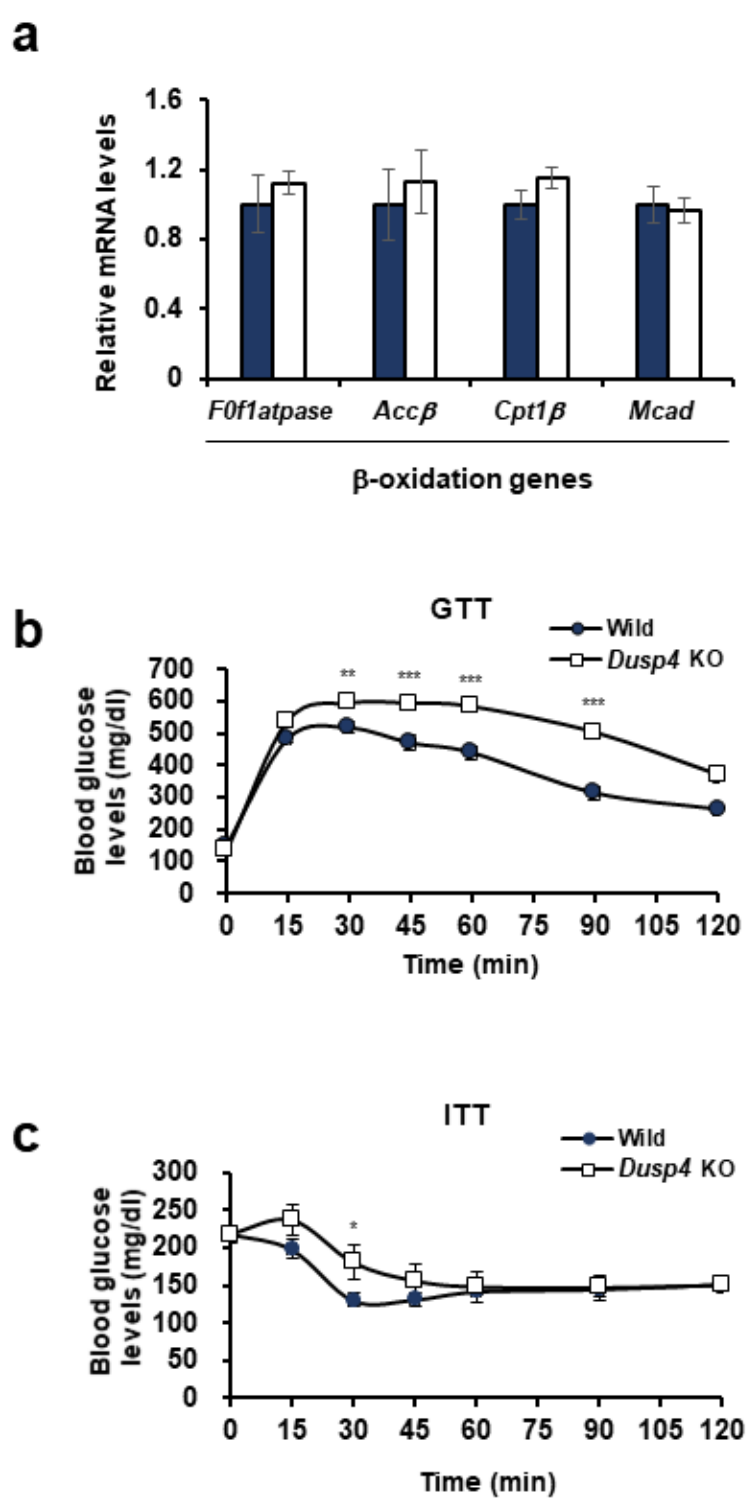

Supple. Fig. 3

Supplementary Fig. 3 Metabolic phenotype of *Dusp4* knockout mice under a high-fat diet (HFD). **a** Relative mRNA expression of key metabolic genes was measured by real-time PCR in iWAT of *Dusp4* KO and wild-type (WT) mice after 10 weeks of HFD feeding (n = 5–8 per group). **b**, **c** Glucose tolerance test (GTT) and insulin tolerance test (ITT) were performed to assess systemic insulin sensitivity in HFD-fed *Dusp4* KO and WT mice (n = 10 per group). *Dusp4* KO mice showed impaired glucose clearance compared with WT controls, as assessed by the glucose tolerance test (GTT) (**b**). In contrast, insulin tolerance test (ITT) results revealed no significant differences (**c**). Data are presented as mean ± SEM. Statistical significance was determined using unpaired two-tailed Student's *t*-test. *$p < 0.01$, **$p < 0.005$, ***$p < 0.0005$.

Supplementary Fig. 4

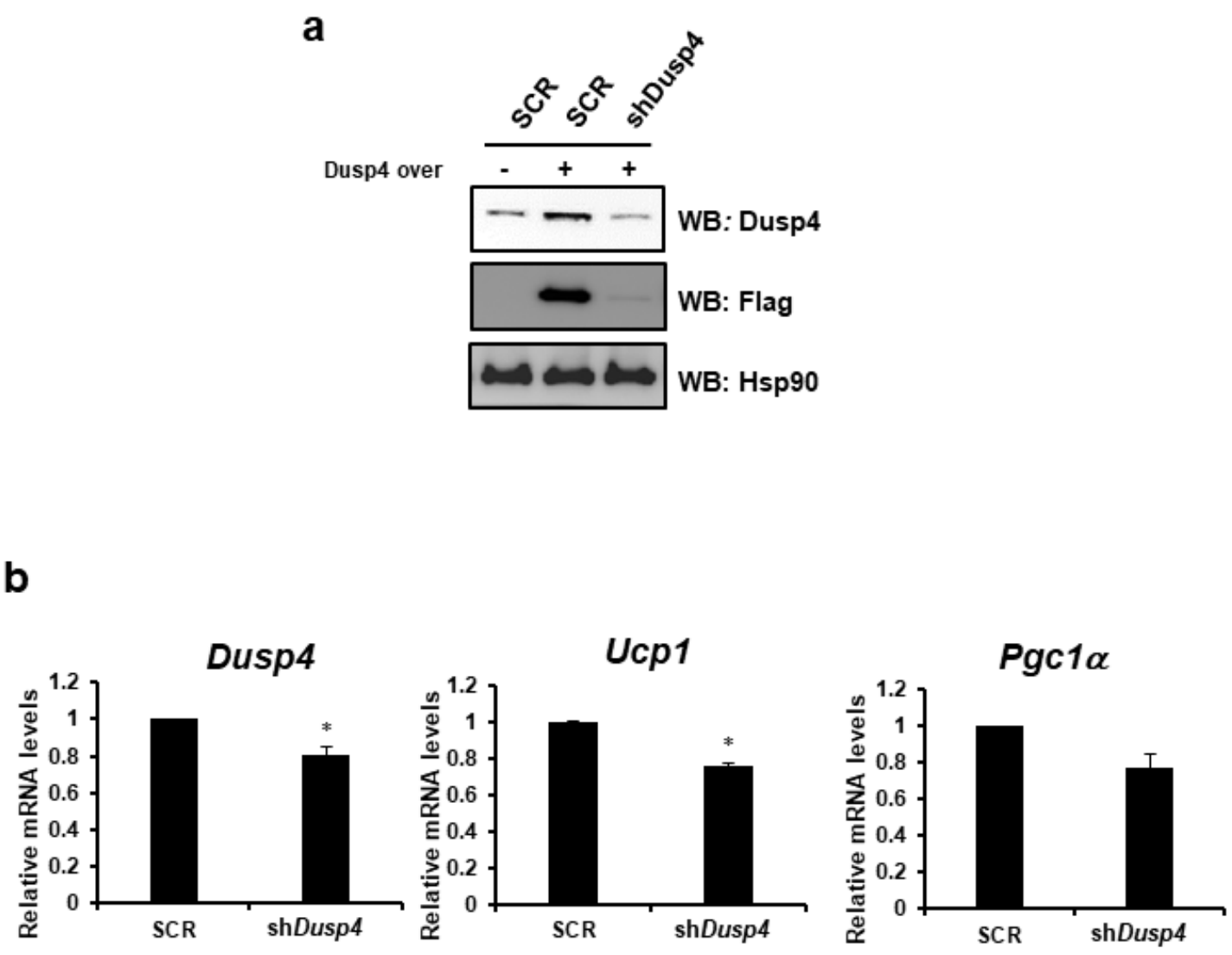

Supple. Fig. 4

Supplementary Fig. 4 Knockdown of *Dusp4* decreases thermogenic gene expression in adipocytes. To investigate the cell-autonomous role of *Dusp4* in the regulation of thermogenic genes in adipocytes, mature inguinal white adipocytes were subjected to *Dusp4* knockdown followed by NE stimulation to induce a thermogenic response. **a** Western blot analysis confirmed a reduction in Dusp4 protein levels upon knockdown in HEK293T cells. **b** Quantitative real-time PCR further demonstrated that knockdown of *Dusp4* significantly decreased the mRNA levels of thermogenic genes, such as *Ucp1* and *Pgc1α* in response to NE treatment, relative to control cells (n = 3). These findings suggest that *Dusp4* is required for the full induction of the thermogenic gene program in white adipocytes under adrenergic stimulation. Data are expressed as mean ± SEM. Statistical analysis was performed using a two-tailed unpaired Student's *t*-test; $*p < 0.01$, $**p < 0.005$, $***p < 0.0005$.

Supplementary Fig. 5

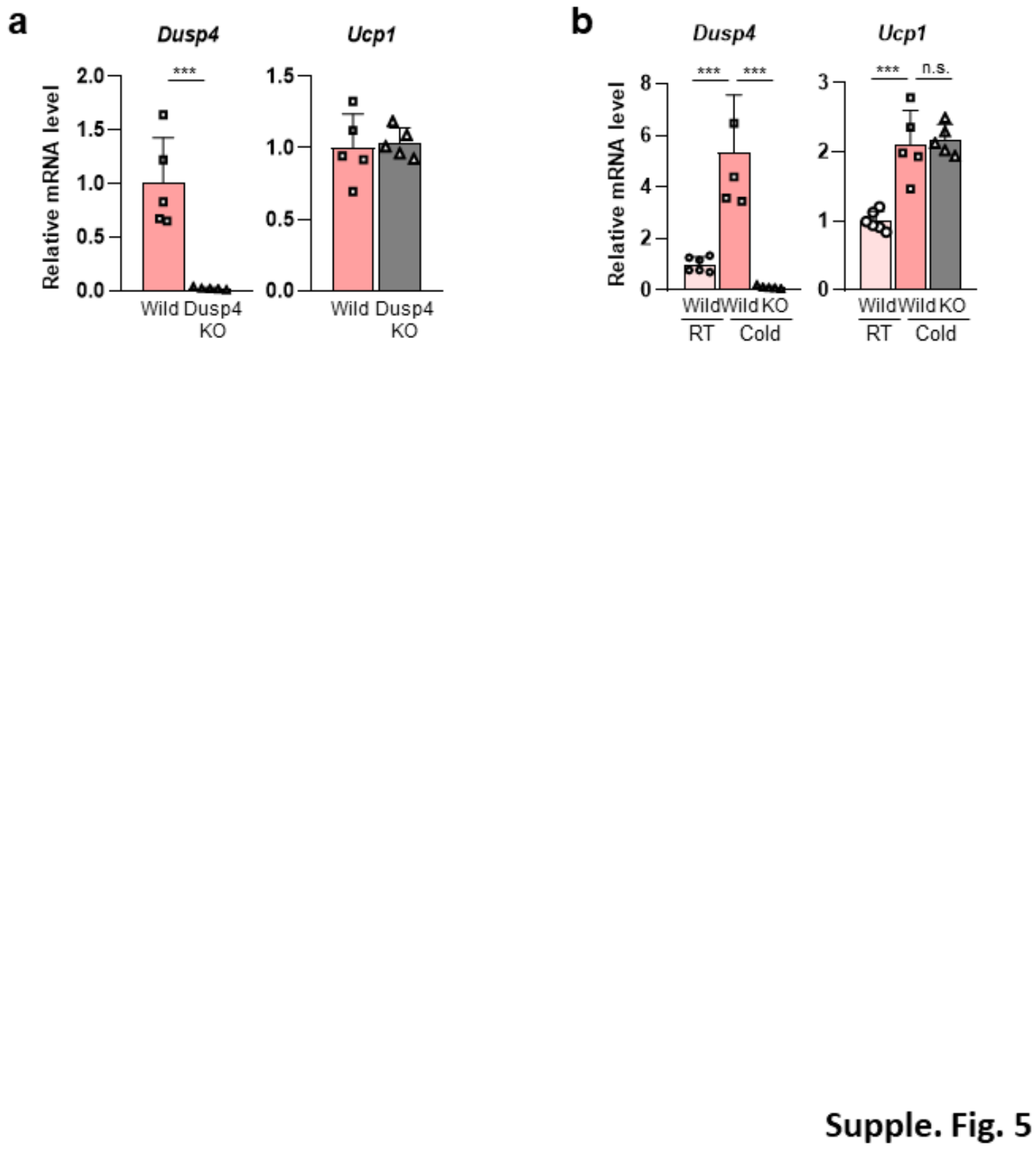

Supplementary Fig. 5 *Dusp4* is dispensable for cold-induced Ucp1 induction in BAT.

a Relative mRNA expression of *Dusp4* and *Ucp1* in interscapular BAT from wild-type (WT) and *Dusp4* knockout (KO) mice maintained on a normal chow diet (NCD). **b** Relative mRNA expression of *Dusp4* and *Ucp1* in the BAT of WT and Dusp4 KO mice housed at room temperature (RT; 22°C) or subjected to cold exposure (cold shock; 6°C). Data are presented as mean ± SEM; each symbol represents an individual mouse. Statistical analysis was performed using a two-tailed unpaired Student's *t*-test; *p < 0.01, **p < 0.005, ***p< 0.0005.

Supplementary Fig. 6

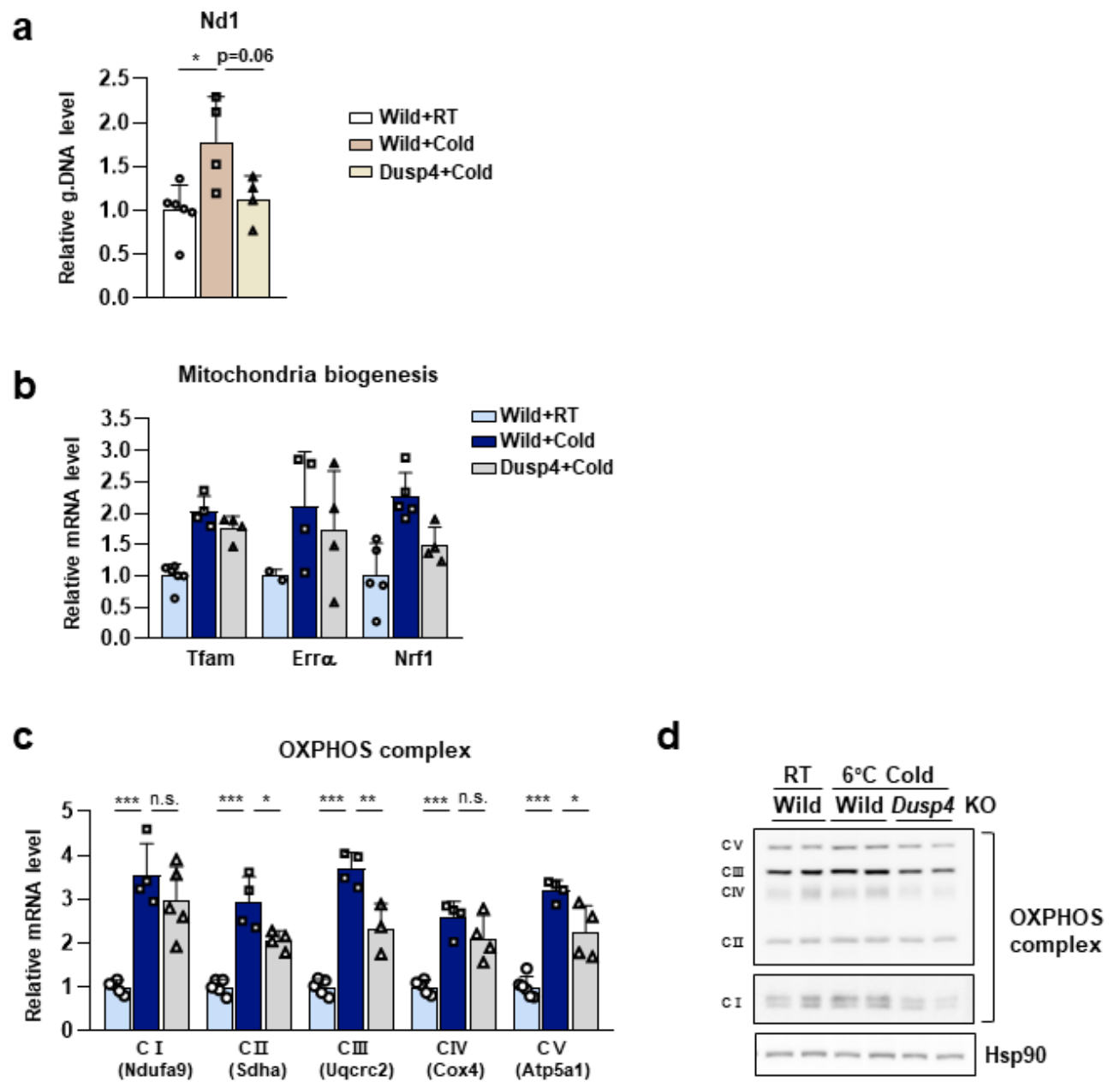

Supple. Fig. 6

Supplementary Fig. 6 *Dusp4* supports cold-induced mitochondrial remodeling in inguinal WAT. **a** Mitochondrial DNA (mtDNA) copy number (mt-Nd1 expression normalized to nuclear DNA) in iWAT from WT and *Dusp4* KO mice housed at RT or exposed to cold (6°C). **b**, **c**, Relative mRNA expression of mitochondrial biogenesis markers (Tfam, Errα, Nrf1) (**b**) and representative respiratory chain/OXPHOS genes (Ndufa9, Sdha, Uqcrc2, Cox4, Atp5a1) (**c**) in iWAT. **d**, Representative immunoblot of OXPHOS complex subunits (CI–CV) in iWAT. Hsp90 was used as a loading control. Data are presented as mean ± SEM; each symbol represents an individual mouse. Statistical analysis was performed using one-way ANOVA with Tukey's post-hoc test (**a**, **b**); *$p < 0.01$, **$p < 0.005$, ***$p < 0.0005$.
